# Preparing Trainees to Rebound from Surgical Complications

**DOI:** 10.1007/s11934-024-01207-7

**Published:** 2024-05-26

**Authors:** Lan Anh S. Galloway, Amy N. Luckenbaugh

**Affiliations:** Department of Urology, Vanderbilt University Medical Center, Nashville, TN USA

**Keywords:** Surgical complications, Adverse events, Surgical trainees, Emotional consequences

## Abstract

**Purpose of Review:**

In this review, we aim to summarize the impact of surgical complications and adverse events on surgeons, including psychiatric illnesses. We evaluate current programs to develop trainee well-being and investigate research within the field of urology.

**Recent Findings:**

Surgical complications and adverse events affect all surgeons, including surgical trainees. Research estimates that 80% of healthcare professionals have been involved in an event that affected them emotionally. These events can affect physicians in many ways, ranging from negatively impacting their quality of life to leading to psychiatric disorders such as acute stress reactions and post-traumatic stress disorder. Unfortunately, there is no standardized preparation to equip trainees to manage and rebound from the profound emotional impact of surgical complications.

**Summary:**

Data in this realm is insufficient, especially in urology, and we need more research in order to better evaluate emotional implications of complications on trainees and how we can prepare trainees to handle them.

## Introduction

Every surgical trainee is bound to experience a surgical complication or adverse event in their residency and career. It is well-known that complications can lead to burnout, decrease in surgeon quality of life, increased anxiety, and in severe cases, psychiatric disorders, such as acute stress reactions and post-traumatic stress disorder. Importantly, these side effects of providing care are common and deeply impactful [1, 2•, 3]. Currently, there is no standard training or preparation to help United States (US) surgical trainees face the emotional consequences of complications. The majority of research on the impact of adverse events on surgeons and trainees has been performed in the United Kingdom (UK) [1, 2•, 3]. This review aims to evaluate the importance and current practices for preparing trainees for surgical complications both within the US and internationally.

## Defining the Gravity of the Problem

Patient safety events are estimated to happen in one of every seven patients, and an estimated 80% of healthcare professionals have been involved in an event that affected them emotionally [1]. In one study of otolaryngology residents in the UK, 94% of residents reported being involved in surgical complication with 61% of them reporting they had not received enough training on the non-technical aspects of handling complications, such as discussing complications with colleagues, confidence, and resilience [1]. Of those residents, 86% believed that coaching on handling complications would be helpful. These residents did have access to an online portfolio tool for reflecting on various events throughout their training, but 81% of residents felt this was not helpful in these scenarios [1]. This study clearly demonstrates the need for further formal education in the sphere of handling surgical complications.

Another study investigating the effect of adverse events on attending surgeons in the UK demonstrated similar findings. Four hundred and forty-five surgeons were included and asked questions regarding recent errors or complications [2•]. Errors were defined as “preventable events arising from shortfalls in the standard of care expected”, and complications were defined as “acknowledged risks of surgical care” [2•]. Forty four percent (44%) of surgeons reported increased anxiety, 43% reported sleep issues, 32% reported anger or irritability, 12% reported increased depression, and 11% reported increased alcohol consumption as a result of an error or complication [2•]. Sleep problems, anxiety, and alcohol consumption scores showed a statistically significant increase after an adverse event [2•]. Notably, this survey polled surgeons on the impact of both errors and complications and noted that outcomes were worse with known errors when compared to complications.

The study also examined coping mechanisms used by physicians in these scenarios. Forty three percent (43%) of physicians who participated in this study did not speak to anyone about the event after it happened [2•]. Among the 57% of surgeons who did speak to someone else about the event, 83% spoke to a colleague and 58% spoke to their partner or friends [2•]. Importantly, most participants did not seek formal support services and surgeons did not feel prepared for these events. When asked to rank how much their training prepared them for the impact of an adverse event personally on a scale of one to seven (with one being “not at all prepared” and seven being “well prepared”), the average score was roughly 3 [2•]. However, when asked if training *should* prepare surgeons for handling the personal impact of adverse events on a scale of one to seven, the average score was 6.2 for all participants [2•]. These studies highlight the immense emotional impact that adverse events can have on surgeons, and the lack of formalized support that is currently available.

## Evaluating for Psychiatric Illnesses in Surgical Residents

Literature shows that post-traumatic stress disorder (PTSD) is seen commonly in surgeons, including up to 15% of US trauma surgeons, but very few studies have examined the effect of complications on psychiatric illness in surgical residents [3]. Adverse events can lead to acute stress reactions (ASRs) and post-traumatic stress disorder (PTSD), characterized by insomnia, anger, decreased concentration, hypervigilance, and exaggerated startle. ASRs are characterized by symptoms lasting less than one month, and PTSD occurs when these symptoms persist for greater than one month. In one study in the UK, 167 surgical trainees were surveyed regarding their stress symptoms after a “stressful event” at work [3]. These events included massive hemorrhage, cardiac arrest, intra-operative death, severe acute traumatic injury, severe acute pain, amongst other stressful events. Sixteen percent (16%) of participants had symptoms consistent with ASR or PTSD, and nearly two thirds of those had not sought professional support [3]. Trainees with symptoms consistent with an ASR or PTSD were more likely to repeat a year of training and were more likely to be female [3].

When asked about whether stress-related symptoms interrupted their training, 31% of respondents reported a minor disruption, 9% reported a moderate disruption, and 4% reported a major disruption [3]. Fourteen percent (14%) of trainees noted they had received pharmacologic or professional support for their mental health [3]. One of the starkest statistics from this study was regarding support after a stressful event - when asked if their program had support services for trainees, 68% of respondents answered “no” or “I don’t know” [3]. Unfortunately, these trainees were poorly equipped to handle these events if the majority reported they either did not know or that there was no support available to them. This is critically important, since as previously stated, stressful patient events can lead to mental health disorders such as ASRs and PTSD.

## Current Programs to Develop Trainee Well-Being

Despite extensive research demonstrating that surgical trainees are not prepared to manage the emotional toll of adverse events, there is no standardized curriculum to prepare trainees for this. Some believe that this kind of preparation should start at the medical student level, and psychiatrists at Wayne State University did just that [4]. Although this was not focused specifically on surgical trainees, but rather an entire medical school class, these researchers designed an educational session for medical students to discuss the impact of errors with physicians in practice [4]. This was an hour-long session for students prior to the start of their clinical years with the goals of teaching students about three important factors related to errors and complications [4]. First, the program emphasized the likelihood that they will be involved in a medical error [4]. Second, the program informed medical students about the professional and personal impacts of medical errors [4]. Lastly, the program identified resources and support personnel should they struggle with the emotional effects of a medical error [4]. Students completed surveys before and after the curriculum to assess their growth. There was a statistically significant increase in student awareness and confidence in coping with medical errors after the training [4]. This study demonstrates the feasibility of a short, but effective training session for medical students to better prepare them to handle the personal toll of medical errors.

Perhaps the most robust example of developing a support and educational system for trainee and surgeon support comes from surgeons at Massachusetts General Hospital. This group developed a peer support program to help surgeons manage the emotional trauma that results from surgical complications. They first conducted a survey amongst three major US hospitals that demonstrated that more than 83% of surgeons experienced a psychological burden due to an intraoperative adverse event [5••]. The study also noted that most physicians turned to colleagues in these difficult scenarios given that they may have experienced similar events in the past. As a result, they created a peer support program at their institution using a five-step process. Initially, they developed the conceptual framework and involved multiple parties, including surgical department leadership, morbidity and mortality conference team, the chief quality officer, and risk management [5••]. Of note, the program was considered quality assurance to protect all participants from litigation [5••]. After the framework was developed, surgical trainees and staff were asked to nominate individuals that “they would be most comfortable reaching out to for nonjudgmental support after a challenging surgical case or adverse event” [5••]. One to two members within each PGY level and each surgical division were then chosen to serve as peer supporters. After selection, peer supporters were trained in a 4-hour session which included a literature review on need for physician support, role play with critiques by observers, and education about resources available to physicians in these scenarios [5••]. The fourth step in the process was to identify major adverse events via morbidity and mortality conferences, mortality review and safety reports, division chiefs and quality directors, and word of mouth [5••]. Finally, they designed a plan for intervention [5••]. Affected physicians were paired with peer supporters and all meetings were considered “opt-out,” meaning that physicians were assigned a peer supporter automatically, unless they declined support [5••].

A year after the program’s creation, participants were surveyed. There were a total of 47 meetings, with 81% of surgeons accepting this support [5••]. Among those that did have peer support meetings, most provided positive feedback. The majority of surgeons either agreed or strongly agreed that their peer “provided a safe and trusting environment for discussion,” was non-judgmental, was an attentive listener, and helped them feel better [5••]. Seventy one percent (71%) of participants either agreed or strongly agreed with the statement that they were satisfied with their experience, and 80% agreed or strongly agreed that they would be likely to recommend this program to a colleague [5••]. Overall, this program provides an excellent example of offering effective support for trainees and surgeons dealing with the emotional toll of adverse events. Surgical departments around the country should consider implementation of similar programs to help mitigate the effects that complications can have on both trainees and supervising surgeons.

## Examining Urology Trainees

Very few studies have investigated the impact of surgical complications specifically on urology residents. However, one recent study in Ireland looked specifically at urologic trainees and aimed to evaluate the emotional effect of adverse events and perceived benefit of support systems [6•]. Participants were also screened for PTSD with a validated primary care tool called the PC-PTSD-V [6•]. Sixty nine percent (69%) of residents described physical or emotional effects after an adverse event [6•]. Reported symptoms included anxiety, guilt, anger, irritability, low mood, sleep problems, impact on personal and professional relationships, cardiovascular symptoms, and gastrointestinal problems [6•]. Sixty three percent (63%) of residents with symptoms endorsed more than one symptom [6•]. Similar to prior studies, the most common coping strategy employed by these urology trainees was discussing the event with someone else, done in 81.3% cases [6•]. Residents most commonly turned to their work colleagues (92.3%) and some turned to friends and family (7.7%), but no respondents reported seeking support services [6•].

Not surprisingly, these urology trainees did not feel prepared for the personal impact of complications. When asked to rate their preparedness on a scale of 1 (not prepared at all) to 5 (well prepared), the median score was 3 [6•]. This study allowed free text responses and multiple residents commented that training on managing complications was “minimal or non-existent”, and that training did not emphasize “how we deal with complications personally” [6•]. Furthermore, on a Likert scale of 1 (completely disagree) to 5 (completely agree), 100% participants agreed that training should better prepare them to manage the effects of adverse events [6•]. Sample size of this study was notably small with only 16 respondents, but this is relatively unsurprising given the specificity of the potential respondent pool. This study is the first to survey urologic trainees specifically and assess their responses to surgical complications. These findings in urology are not unique and mirror the data in general surgery.

## Conclusions

Complications and errors are incredibly common and impactful for surgeons throughout their career as demonstrated in the multiple studies mentioned above. They can lead to increases in anxiety, depression, increased substance use, anger, irritability, and psychiatric disorders, such as acute stress reactions and PTSD. Despite the frequency with which physicians encounter these scenarios, there is no formal or standardized method for preparing trainees for this. A handful of institutions have started to develop methods to prepare their students and trainees to handle these situations, including medical student courses and peer support programs. One of the most encouraging and exciting examples of a trainee support system to help combat the negative effects of complications comes from Massachusetts General Hospital. Not only were they able to develop and implement a peer support program, but importantly, participants viewed it as beneficial for their wellness and something they would recommend to their colleagues.

Data regarding preparing trainees for complications remains scarce in the field of Urology, especially Urology residents in the US. More research is needed to better evaluate the impact of complications on trainees in our field and how we are preparing trainees for that impact. In the meantime, we can extrapolate from our colleagues in other fields and should work towards accruing more data in urology.

## Data Availability

No datasets were generated or analysed during the current study.
